# All-Si Photodetectors with a Resonant Cavity for Near-Infrared Polarimetric Detection

**DOI:** 10.1186/s11671-019-2868-3

**Published:** 2019-01-30

**Authors:** Bo Feng, Jingyuan Zhu, Chen Xu, Jing Wan, Zelong Gan, Bingrui Lu, Yifang Chen

**Affiliations:** 1 Fudan University, Shanghai, China; 2 JiHua Laboratory, Foshan, China; 3 Nanolithography and Application Research Group, State Key Lab of Asic and System, Fudan University, Shanghai, China

**Keywords:** All-Si photodetector, Internal emission of hot electrons, Surface plasmonic resonator, Polarimetric detection, Nanofabrication

## Abstract

This work developed an all-Si photodetector with a surface plasmonic resonator formed by a sub-wavelength Au grating on the top of a Si-nanowire array and the same one beside the wires. The Au/Si interface with a Schottky barrier allows the photo-electron detection in near-infrared wavelength based on the internal emission of hot electrons generated by the surface plasmons in the cavity. Meanwhile, the Au sub-wavelength grating on the Si nanowire array acts as a polarizer for polarimetric detection. Finite-difference time-domain method was applied in the design of the novel device and state-of-art nanofabrication based on electron beam lithography was carried out. The characterization of the photo-electronic properties as well as the polarimetric detection demonstrate that the fabricated detectors on the silicon substrate possesses great prospects for sensing technology on all-Si.

## Background

With the fast advances in optical communication, there is a growing need to develop polarimetric photodetectors (PDs) in the near-infrared (NIR) wavelength at low cost. Although III-V compounds such as GaAs/InGaAs and II-VI ones such as TeCdHg have been the most successful option for PDs in the past decades due to their relatively large absorption coefficients [1–5], the complexity in growth and the high cost in manufacturing are always the biggest issue for general applications. Especially, there is still a long way to go before polarimetric detection is realized by the PDs in III-Vs and II-VIs. Being the major material of the semiconductor industry, silicon has emerged as optoelectronic devices in recent years due to their distinct optical and electrical properties [6–8], well-established process, and high compatibility with the developed CMOS technology [9]. Furthermore, recent achievements in silicon photonics [10, 11] offer a promising pathway to realize the novel form of PDs by integrating Si nanowire detectors [12, 13] with photonic structures for new application such as polarimetric detection.

Based on our earlier success in developing Si nanowire (Si NW)-based PDs [12], this paper further proposes a new form of all-Si photodetectors by integrating subwavelength metallic grating with silicon nanowires to achieve polarimetric detection in near-infrared (NIR) wavelengths. To fulfill this task, the following three issues need to be resolved. First, conventional Si nanowire-based PDs work in visible wavelengths (0.4–0.7 μm), it is essential to drive the Si nanowire detectors into NIR regime [13, 14]. Secondly, a miniaturized optical polarizer needs to be built into the detector for polarimetric detection. Thirdly, owing to the low absorption coefficient of Si in NIR, light harvest structure is desired to enhance the responsivity. To meet all these requirements, this work has developed a novel device structure in silicon, which is composed of subwavelength metallic grating as a polarizer, silicon nanowire array with certain height for light harvest, and finally, a surface plasmonic resonator for wavelength selection and for the emission and diffusion of hot electrons [15–20] over the Schottky barrier in the Au/silicon interface to generate an extra photocurrent under illumination. This resonant cavity-based strategy not only extends the band edge of Si into the IR regime but also broadens the bandwidth of the photoresponse with polarization-sensitive detection. This paper reports our recent progress in tackling all these issues.

## Methods/Experimental

### Design of the All-Si Polarization Detectors

**Fig. 1:**
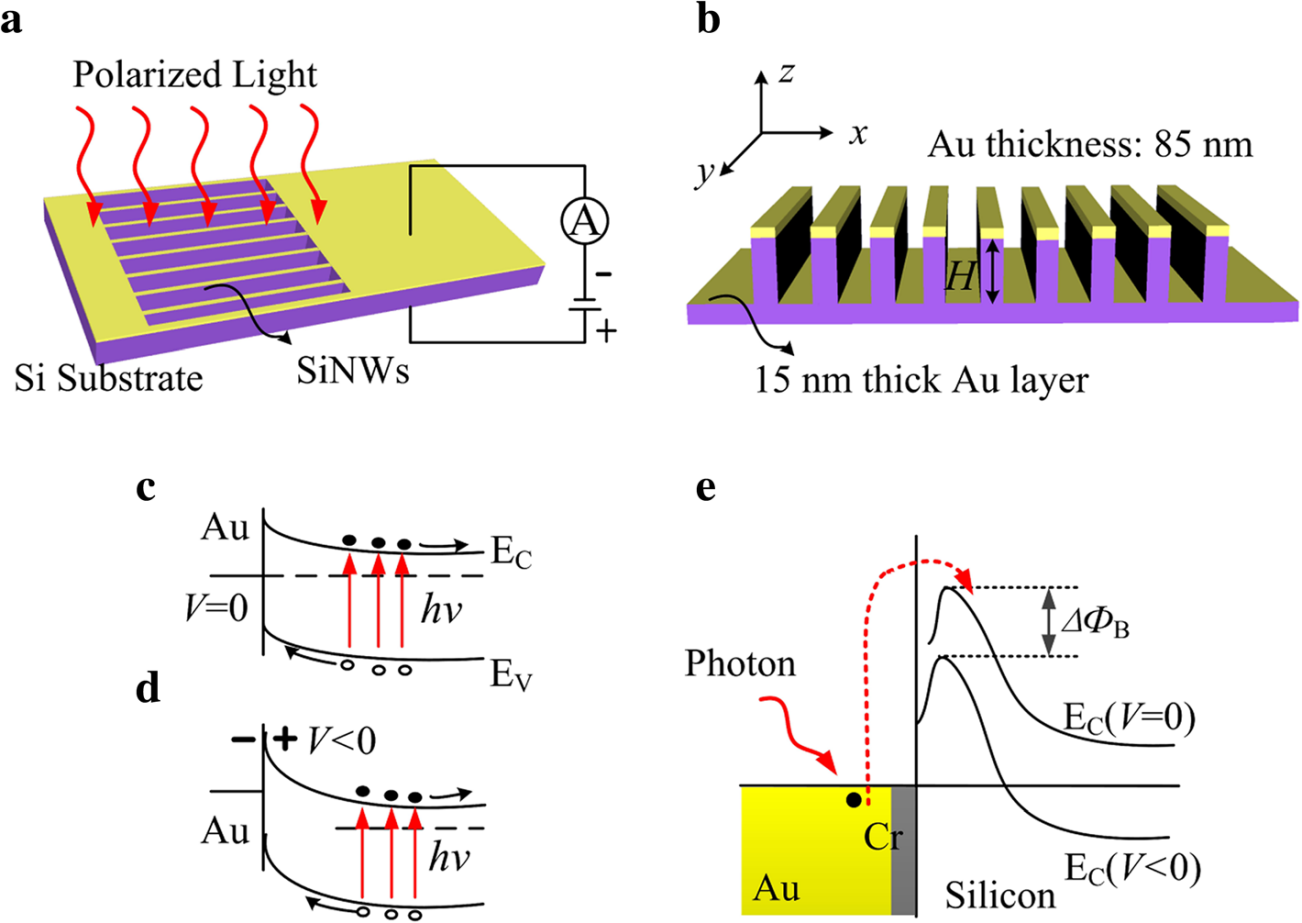
Schematic diagram of the resonator tuned MS photodetector in silicon and its photo-electronic principle. **a**, **b** The diagram of the detector. **c**, **d** The energy band for simple MS junction under IR illumination with and without the DC bias. **e** The diagram showing the internal emission of hot electrons from surface plasmons

Figure 1a is the schematic diagram of the device. Si nanowire arrays with the pitch of 400 nm and the heights (*H*) from 100 nm to 300 nm were fabricated on lightly n-type doped silicon substrate (500 μm thick, 1–10 Ω cm) by a conventional dry etching process. A Schottky barrier was established in the metal grating-semiconductor (MS) interface. Figure 1b shows a surface plasmonic resonator between the top and the bottom metallic layer, surrounding the Si NW.

Figure 1c and d are the diagrams for the band bending in Si near the MS interface under illumination without or with a DC bias, respectively. The optoelectrons were generated only when the photons’ energy satisfies *hν* > *E*_*g*_, where *h* is the Planck constant and *E*_*g*_ is the Si bandgap, corresponding to the detection in visible wavelengths. However, as shown in Fig. 1e, hot electrons generated through the internal photoemission effect (IPE) [10, 11, 15] by surface plasmons in the metallic layer can diffuse to the Si substrate and flow over the Schottky barrier as the extra photo-current, enabling the detection in NIR. Furthermore, in this scenario, the subwavelength Au grating on the nanowire top acts as a polarizer as well as a resonator tuning the detecting wavelengths, determined by the dimensions of the structure.

### FDTD Simulations

In order to optimize the device structure for polarimetric detection with high quantum efficiency in NIR wavelengths, a 3D finite-difference time-domain (FDTD) simulation study using Lumerical software package was systematically carried out. In the simulation, the periodic boundary condition along *x* and *y* and perfectly matched layers along the *z* direction were adopted. A plane wave with the TM mode in parallel to the *x*-axis, acting as the optical stimulation source, propagated along the z direction. The thickness, the width, and the pitch of the Au grating are set to be 85 nm, 200 nm, and 400 nm, respectively. A reflection monitor was placed at the top of the simulation region and a transmission monitor was placed at the bottom of the Si substrate. The optical absorption spectra of the device were obtained from the measured reflection (*R*) and transmission (*T*), using *A* = 1-*R*-*T*.

### Device Fabrication

Nanofabrication for the as-designed metal/semiconductor photodetector was carried out using electron beam lithography based process. On the n-type silicon (1–10 Ω cm, < 100 > orientation), a 300-nm thick PMMA supplied by Micro-Chem Ltd. was first spin-coated, followed by a soft-bake on a hot plate for 12 min at 180 °C. After the e-beam exposure by the beam writer of JEOL 6300FS, the exposed resist was developed in a MIBK/IPA (1:3) solution at 23 °C for 60s, finished by a thorough rinse in IPA solution for 15 s. A wet etch in 2%-buffered HF was applied to remove the native oxide on silicon. The samples were immediately transferred into a thermal evaporator for the deposition of 2-nm Cr/70-nm Au. The 2-nm Cr is crucial for determining the Schottky barrier height and adhering the Au gratings to the silicon. The unwanted material was then removed by lift-off in acetone at 60 °C. The sample was finally rinsed in ample isopropanol and dried with compressed N_2_. By this stage, a large bonding pad with a square window was formed. Then, the top electrode appearing as a subwavelength grating in Cr/Au was laid down in the square window and connected with the pad, using registration technique, through the same process as described above. Using the patterned metallic structure as etching mask, a reactive ion etch (RIE) in fluorine-based plasma was carried out in a Samco etcher to form Si nanowires. Finally, a 15-nm Au film was deposited on to the whole device to form a resonant cavity, as illustrated in Fig. 1b.

### Photoelectric Characterization

Photo-electronic properties of the fabricated detectors were systematically characterized in the wavelength of 0.7–1.1 μm using a conventional optoelectronic response setup. The light source was calibrated by a power meter, supplied by OPM 35S Ltd.

## Results and Discussion

**Fig. 2:**
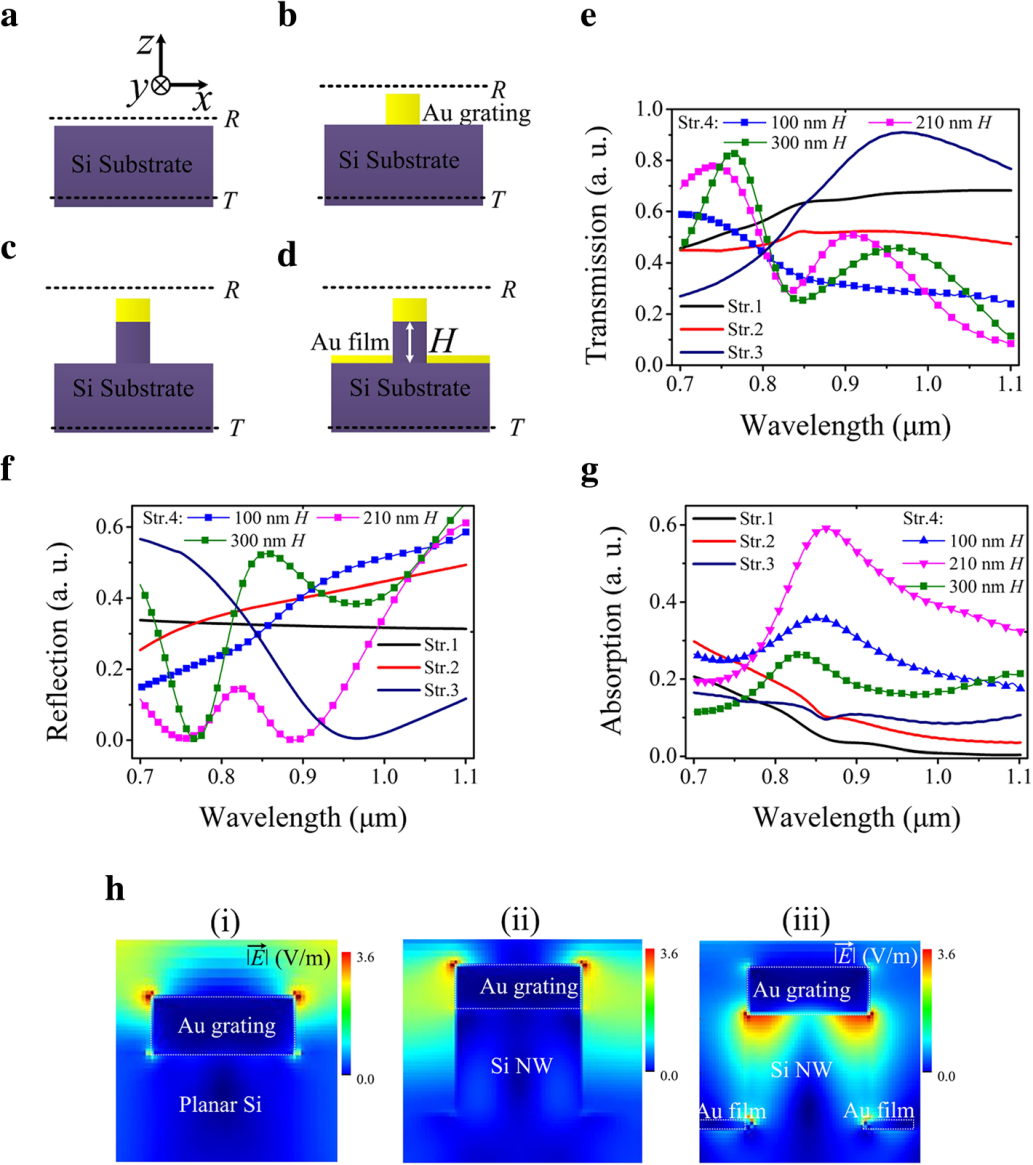
The diagrams for the four devices investigated in this work and the FDTD simulation results from the devices. **a** Str.1: the planar Si substrate. (**b**) Str. 2: the Au grating on the top of Si substrate. (**c**) Str. 3: the Au grating on the top of Si NW array. (**d**) Str.4: the completely fabricated detector with both the Au grating on the top and the bottom of the Si NW array. **e**–**g** The simulation results for the transmission, reflection, and absorption spectra through the four structures, respectively. **h** The simulation results for the electric field distributions in the three structures as depicted in **b**, **c**, and **d**, respectively, and the wavelength of the incident light is 860 nm

Figure 2a–d depict schematically the 2D cross section of the device structure. To understand the working mechanism, four kinds of device structures, a planar Si surface surrounded by a bonding pad on a Si substrate (Str. 1 in Fig. 2a), a Au grating on Si surface (Str.2 in Fig. 2b), a Au grating followed by 210 nm-*H* Si NW (Str.3 in Fig. 2c), and a resonator tuned device (Str.4 in Fig. 2d) were compared. The simulated spectra for the transmission, reflection, and absorption are shown in Fig. 2e–g, respectively. The electric field distributions in the device with the Si NW height of 210 nm were calculated for the light at the wavelength of 860 nm. Figure 2h (i–iii) show the results for the device Str.2, Str.3, and Str.4 respectively.

The simulation results presented in Fig. 2e and g depict an extremely interesting picture for the light transmission/absorption process in the proposed photodetector in the wavelength of 0.7–1.1 μm. While this device was illuminated by TM-polarized light (E-field perpendicular to the NW direction), the transmissions through the planar silicon for the Str.1 (Fig. 2a) is mostly above 50%, corresponding to low absorption by Si as expected. The addition of an Au grating to the planar silicon surface, as shown in the structure Str.2 (Fig. 2b), just leads to a 10–20% reduction in the transmission. For the photodetector structure (Str.4) as illustrated in Fig. 2d, the transmissions in 0.7–0.8 μm are significantly enhanced, even beyond those through the planar silicon (the reason still needs to be investigated). However, the more striking feature is that the transmission and reflection (Fig. 2f) in the wavelengths of 0.825–0.875 μm is considerably reduced for 210 nm-*H*, and the absorption is boomed well above those in the other structures. The physical picture behind such an increase in the absorption can be interpreted by the resonant modes in the Fabry-Perot cavity formed by the two metals on the top and the bottom beside the Si nanowires. The high electric field existing between the top and the bottom Au layers, as presented in Fig. 2h (iii) by the FDTD simulation at 860 nm of the wavelength, stands for the resonant modes of surface plasmons. It is believed that the absorption of the resonant energy was converted to generate hot electrons in the metallic layers via plasmon decay at high efficiencies. Such a remarkable absorption characteristic lays a solid foundation for the novel photo-electron detection in NIR by the designed Au/Si Schottky barrier detector. Especially, Fig. 2g also shows that a resonator tuned photodetector exhibits an absorption with full width at half maximum (FWHM) up to 300 nm.

**Fig. 3:**
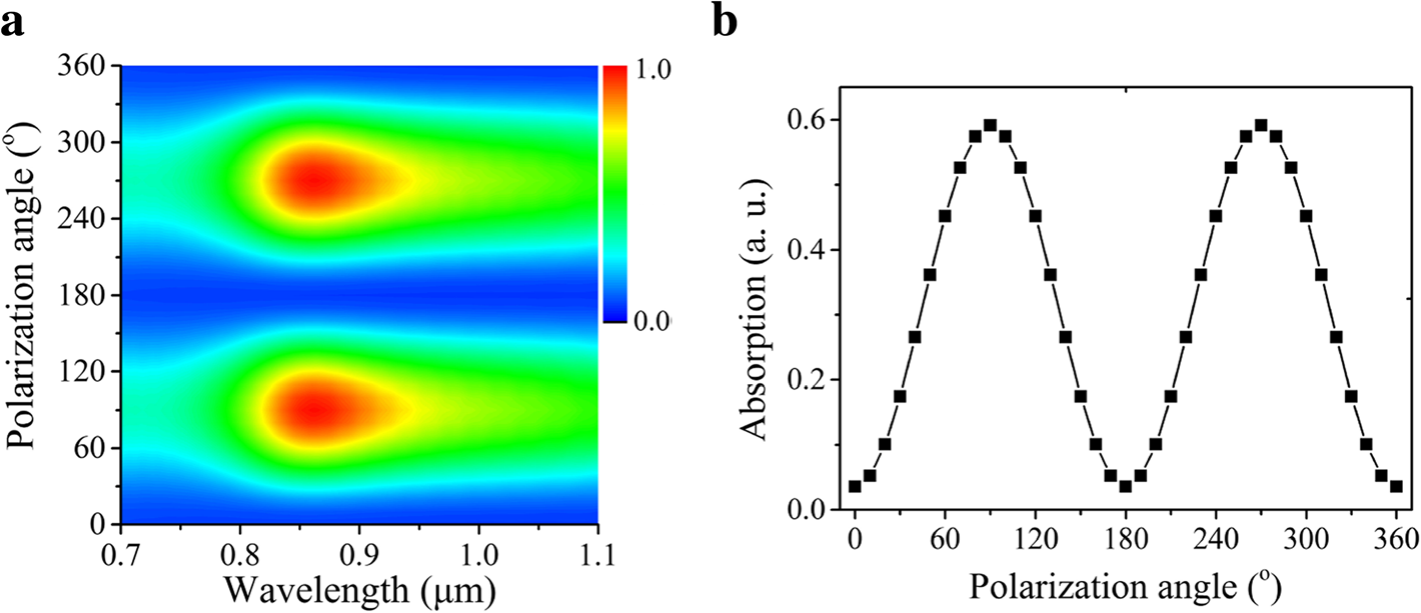
The theoretical results for the polarization properties of the photodetector with the surface plasmonic resonator. **a** The polarization dependence of optical absorption spectra at different polarization angle. The 0°of polarization angle was defined along the direction of the Au grating. **b** Polarization-dependent absorption intensity with incident wavelengths of 860 nm

Furthermore, for polarization detection, the sub-wavelength metallic grating on the top of Si nanowires is also a polarizer, converting the incident light into TM polarized. The polarization characteristics were also studied by calculating the absorption spectrum for the designed resonator structure in Fig. 2d. Figure 3a presents the angle-dependent absorption spectra in 0.7–1.1 μm when the nanowire height (*H*) was 210 nm, where 0° corresponds to the polarization in parallel to *y*-axis. The 3D plot of wavelength-polarization angle-absorption in Fig. 3a indicates the maximal absorption happen at the wavelength of 860 nm, which is consistent with the peak position in Fig. 2g. The strictly periodic variation of the absorption with the polarization angle in Fig. 3b gives rise to the extension ratio (peak/valley) of ~ 17:1. To further enhance this ratio, the grating profile needs to be optimized.

**Fig. 4:**
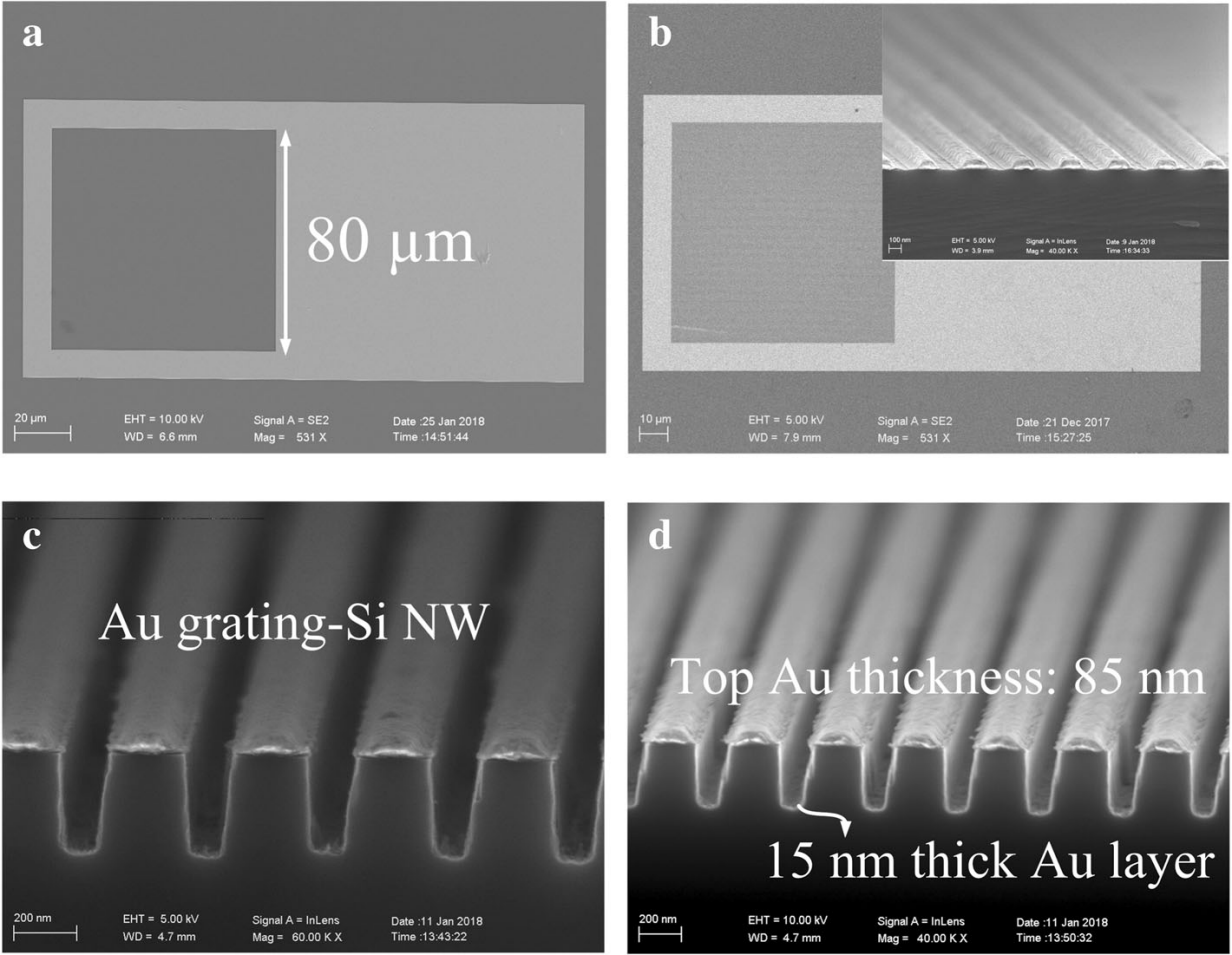
Micrographs by scanning electron microscope (SEM) for the fabricated MS photo-electron detectors. **a** Str.1: the overview of the device with the bonding pad only. **b** Str.2: the Au grating-planar Si located inside the square window. **c** Str.3: the cross-sectional view of Au grating-Si NW device. **d** Str.4: the cross-sectional view of the finally fabricated device with resonant cavities

Figure 4 shows the fabricated four kinds of structures: the bonding pad on a planar Si substrate with a square window (Fig. 4a), the Au grating-planar Si registered in the square window (Fig. 4b), the Au grating-Si NW device (Fig. 4c), and the final resonator tuned device (Fig. 4d), respectively. The overall dimensions of the device layout from the top view is 200 μm × 100 μm, and the square window measures 80 μm × 80 μm. In correspondence to the design, the Au-grating lines and spaces are 200 nm and 400 nm, respectively. Annealing of the devices in nitrogen gas at 350 °C for 10 min was undertaken, aiming to reduce the surface defects on the nanowires [21, 22].

**Fig. 5:**
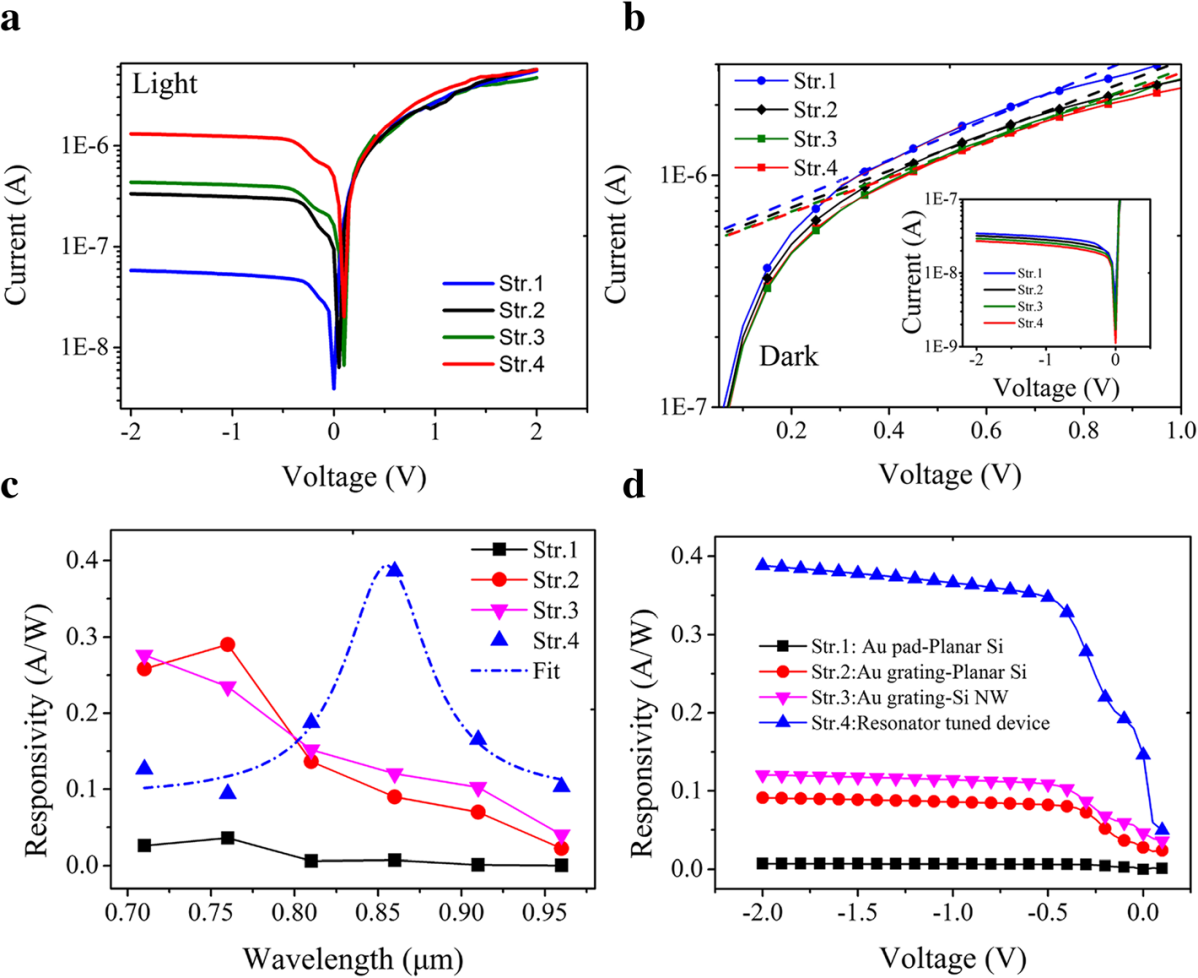
The measurement results obtained from the fabricated all-silicon detector. **a** Light logarithmic *I*-*V* curves under the illumination intensity of 16.6 mW/cm^2^. **b** Dark logarithmic *I*-*V* curves. **c** Responsivity spectra under the bias of − 2 V and the light intensity of 16.6 mW/cm^2^. **d** Bias dependency of responsivity for 860 nm wavelength under the intensity of 16.6 mW/cm^2^

Figure 5a depicts the current-voltage (*I*-*V*) curves taken from the four different devices under illumination of 16.6 mW/cm^2^ at 860 nm wavelength, respectively. Under the negative DC bias from the top electrode to the silicon substrate, surface plasmonic resonator-based photodetector (Str.4) with 210 nm-*H* demonstrates an increase of the current by an order of magnitude, which is the highest photocurrent among the four devices, despite the current flow in the positive bias coincide with each other. Compared to Au grating-Si NW device (Str. 3), the resonator-tuned device (Str.4) realizes a larger current under illumination, which reveals the existence of an extra photo-current caused by the additional metallic film architecture (Fig. 1e).

The *I*-*V* characteristics in dark are further analyzed using the thermionic emission model [10, 23]. The thermionic emission current is given by:$I={AA}^{\ast }{T}^2\exp \left(-\frac{q{\Phi}_B}{kT}\right)\left[\exp \left(\frac{qV}{nkT}\right)-1\right]$, where *A* is the area of the contact junction, A* is the Richardson constant (≈ 112 A cm^−2^ *K*^−2^ for n-type Si), *T* is the temperature, Φ_B_ is the Schottky barrier height, *k* is the Boltzmann constant, *q* is the electronic charge, *n* is the ideality factor, and *V* is the voltage drop across a junction. The Φ_B_ and *n* can be extracted via linear fitting of lg *I*-*V* in the forward bias linear region, as shown in Fig. 5b. The *q*Φ_B_ and *n* for resonator tuned device (Str. 4) are found to be 0.57 ± 0.016 eV and 1.43 ± 0.028 with an adjusted *R*^2^ of 0.99644, respectively. The ideality factor is closed to 1, which indicates the thermionic emission is the main current mechanism. The reverse bias behavior (− 2, 0) is shown in the inset of Fig. 5b, which shows the lowest dark current (~ 27 nA) achieved in Str.4. Two factors may help to reduce the dark current: one is the increasing of nanowire resistance, and the other is the conductivity decrease, due to a thin interface depletion layer between 15-nm-thick Au layer and silicon.

It is well known that the responsivity (*R*_λ_) is a critical parameter for optical devices, which can be defined as *R*_λ_ = *I*_ph_/*PS*, where *I*_ph_ is the photocurrent (*I*_Light_-*I*_Dark_), *P* is the illumination intensity, *S* is the overall photoelectronic sensing area, which is the actual area of all the layout measured from top view [12]. As presented in Fig. 5c, the responsivity spectrum by the resonant cavity based photodetector (Str. 4) shows the maximum of 0.386 A W^−1^ around the wavelength of 860 nm and a FWHM of 150 nm under the bias of − 2 V. Such a peak responsivity agrees with the maximum absorption simulated by the FDTD method as shown in Fig. 2g. These results again demonstrate the existence of plasmonic hot electrons in the metallic layer. The other three devices, however, give rise to the responsivities of 0.007 A W^−1^, 0.09 A W^−1^, and 0.121 A W^−1^, respectively. More importantly, no peak is observed throughout the wavelengths in 0.7–1.1 μm as concerned. Furthermore, considering a Fowler response [20] modified by the plasmon absorption spectrum *S*(*v*): *R*(*v*) = *η*_*i*_ ⋅ *S*(*v*), and ${\eta}_i\approx {\mathrm{C}}_F\frac{{\left( hv-q{\phi}_B\right)}^2}{hv}$, which describes the number of “available” electrons in the structure with sufficient energy to overcome the potential barrier [24–27]. Based on this, fitting the experimental responsivities of Str. 4 as shown in Fig. 5c by a Lorentzian line shape for S(*v*), a Schottky barrier height of 0.578 ± 0.0127 eV with an adjusted *R*^2^ of 0.94611 was obtained, which is similar to the abovementioned 0.57 eV and indicates the main detection mechanism is IPE. As an added benefit, this resonator-based photodetector provides significant photocurrent tuning through the application of a negative bias to the device, offering a good control of the responsivity, as shown in Fig. 5d. It also shows a considerable responsivity of 0.146 A/W at 0 V bias.

The characterization of the optoelectron response property for the fabricated device demonstrates that the designed photodetector is able to work in the NIR region. The experimental comparison of the photo-electron responsivity between the devices with and without the resonator provides us with a strong evidence for the resonant absorption of the light in NIR, leading to the internal photon emission (IPE) in the Au grating/Si Schottky interfaces. When the generated hot carriers gain sufficient energy to overcome the Schottky barrier, extra photocurrent is collected by the silicon substrate. The measured responsivity, however, is still below the average value comparing with conventional detectors. Further improvement should be made by reducing the top Au layer thickness down to 30 nm so that most of the generated hot electrons are able to diffuse into the silicon, considering the diffuse length of them is ~ 35 nm [16].

**Fig. 6:**
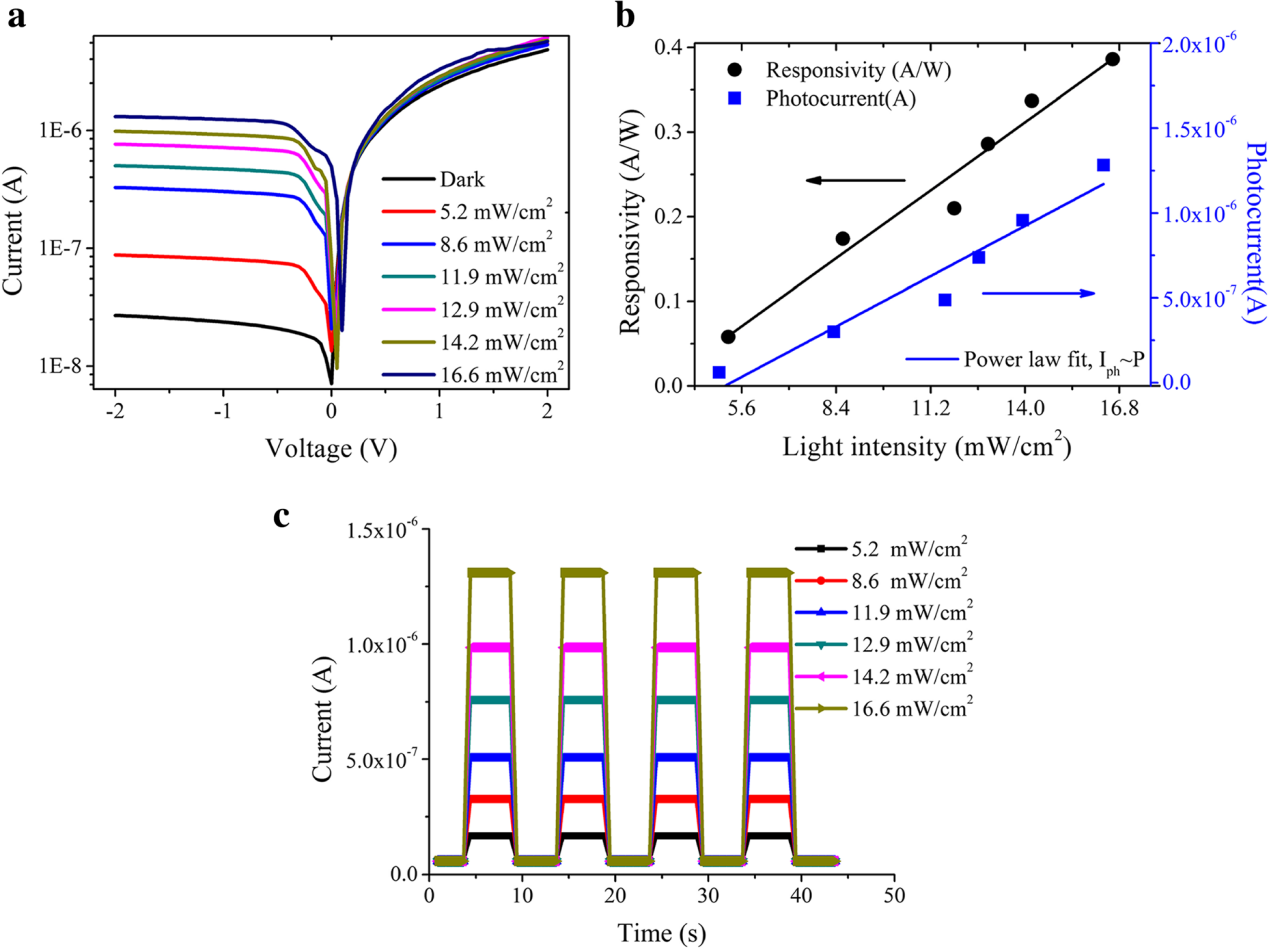
The photo-electron properties of the fabricated detector with the plasmonic resonator. **a** Logarithmic *I*-*V* curves of the detector measured in dark and under different illumination intensities. **b** The curves of the responsivity changing with the illumination intensity under the bias of − 2 V. **c** I-t response of the photodetectors under different illumination intensities at − 2 V bias

Figure 6a presents the measured *I*-*V* curves of the fabricated photodetector with the resonators (Str.4) as illustrated in Fig. 2d under various illumination intensities at the wavelength of 860 nm. Figure 6b show the photocurrent (*I*_ph_) and responsivity (*R*_λ_) as a function of the light intensity under − 2 V. Within the incident light intensity range from 5.2 to 16.6 mW/cm^2^, the photodetector shows a linear response with photocurrent from 6.05 × 10^−8^ to 1.28 × 10^−6^ A, corresponding to responsivity from 0.058 to 0.386 A W^−1^. In Fig. 6b, the solid squares are the experimental data and the solid line is a fit to the simple power law, *I*_ph_ = *AP*^θ^, where *A* is a constant, *P* is the light intensity, and the θ of 1 is an exponent, which confirms that the photocurrent is mostly determined by the amount of photo-generated carriers [28–31]. The photo-electron detection is once again demonstrated by the photocurrent modified by the incident light in square wave form, as shown in Fig. 6c, which shows clear light intensity dependence.

**Fig. 7:**
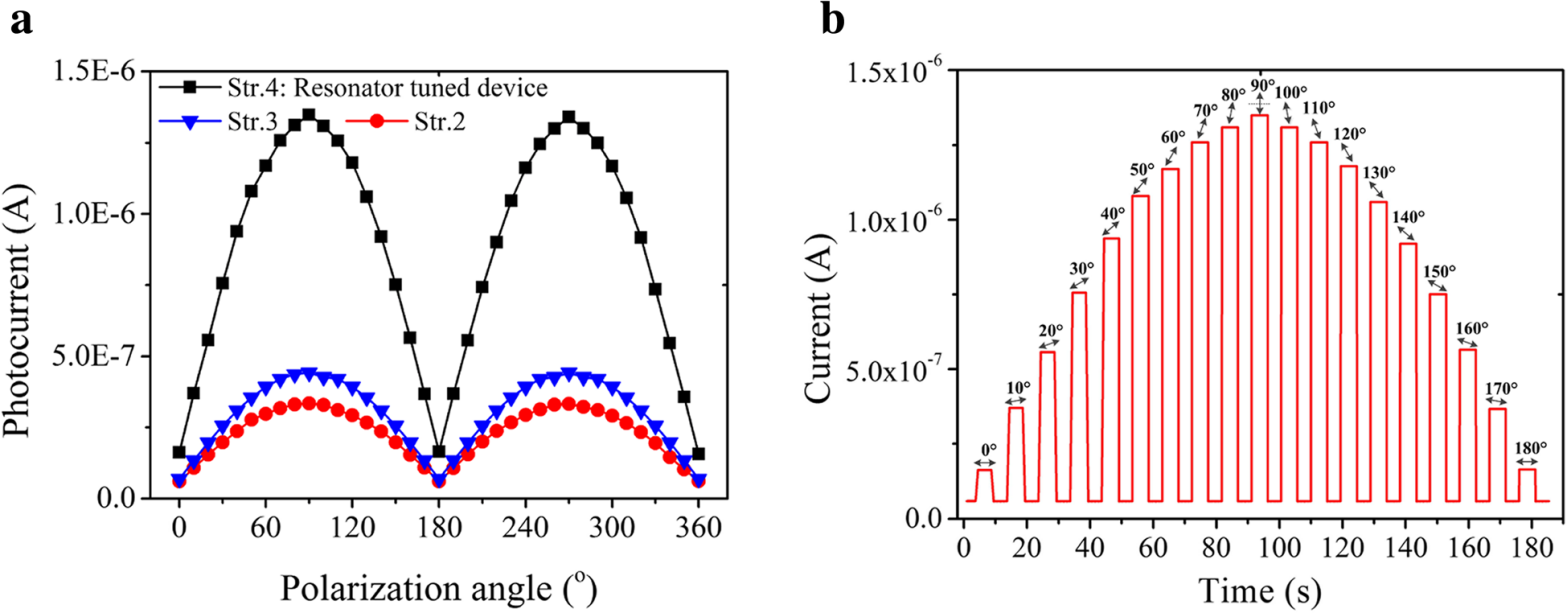
Experimental demonstration of polarimetric detection by the fabricated all-Si photodetector. **a** Polarization dependence of the photo-electon current. **b** Photocurrent response of resonator tuned MS detector under the 16.6 mW/cm^2^ incident light with different polarization angles measured at the DC bias of − 2 V. The polarization angle was marked with a black arrow onto its corresponding photocurrent

The polarization sensitivity of the fabricated Au grating-Planar Si (Fig. 4b), Au grating-Si NW (Fig. 4c), and the resonant cavity tuned device (Fig. 4d) was also characterized using the polarized light of 16.6 mW/cm^2^ under − 2 V bias, as presented in Fig. 7. The photocurrent peak to valley ratios of these three devices are 5.6, 6.4, and 8.3, respectively. It showcases the stronger polarization dependent detection by the all-Si photodetector with the resonant cavity than that with Au grating-Si NW structure. Furthermore, the fast response of photocurrent tuned by the polarization angle is presented in Fig. 7b, demonstrating the polarimetric detection by the fabricated 3D resonator architecture.

## Conclusions

Combining a sub-wavelength grating in Au on silicon as both the etching mask and the polarizer, Si-nanowires as detector material, and a plasmonic resonator formed by a bilayer of Au gratings, this work successfully proposed a novel photodetector based on all-Si nanowire array with polarimetric detection in NIR wavelengths. It was shown that the responsivity of this device was high up to 0.386 A W^−1^ at the DC bias of − 2 V, which is respectively comparable and larger than the values expected for a all-Si IR detector. Furthermore, polarization detection was also achieved and the peak to valley ratio of 8.3 for photocurrent under the incident polarized light at the wavelength of 860 nm was observed. The FDTD simulation of the device performance suggests that the detection wavelength can be tunned in the NIR regime, which is determined by the device structure. Optimization of both the structural dimensions and nano-processing condition will surely improve the extension ratio significantly. The results obtained in this work is instructive to the further development of all-Si nanowire-based polarization detectors toward practical applications.

## Abbreviations

3D: Three-dimensional
DC: Direct current
EBL: Electron beam lithography
FDTD: Finite-difference time-domain
FWHM: Full width at half maximum
IPE: Internal photoemission effect
*I*-*V*: Current-voltage
MS: Metal-semiconductor
NIR: Near-infrared
NW: Nanowire
PDs: Photodetectors
RIE: Reactive ion etch
SEM: Scanning electron microscope
